# Introduction to Editorial Board Member: Professor Mark R. Prausnitz

**DOI:** 10.1002/btm2.10141

**Published:** 2019-08-30

**Authors:** Harvinder S. Gill

**Affiliations:** ^1^ Department of Chemical Engineering Texas Tech University Lubbock Texas

1

In this issue of *Bioengineering and Translational Medicine*, we are delighted to introduce our Editorial Board Member, Prof. Mark R. Prausnitz. Prof. Prausnitz is the Regents' Professor and J. Erskine Love, Jr. Chair in Chemical & Biomolecular Engineering at Georgia Institute of Technology, Atlanta, GA. He received his BS degree (with distinction) in Chemical Engineering from Stanford University and his PhD degree in Chemical Engineering from Massachusetts Institute of Technology.

Prof. Prausnitz is one of the world's leading experts in drug delivery. He harnesses various biophysical methods and phenomenon in clever and unique ways to develop novel methods of drug delivery. In his laboratory, he has invented microneedles for drug delivery, and has used lasers, ultrasound, thermal energy, fluid convective forces, and other approaches to deliver both small and large molecules into tissues and cells. He constantly challenges himself and his laboratory members to develop innovative yet simple and cost‐effective solutions that can make an impact on human lives.

**Figure 1:**
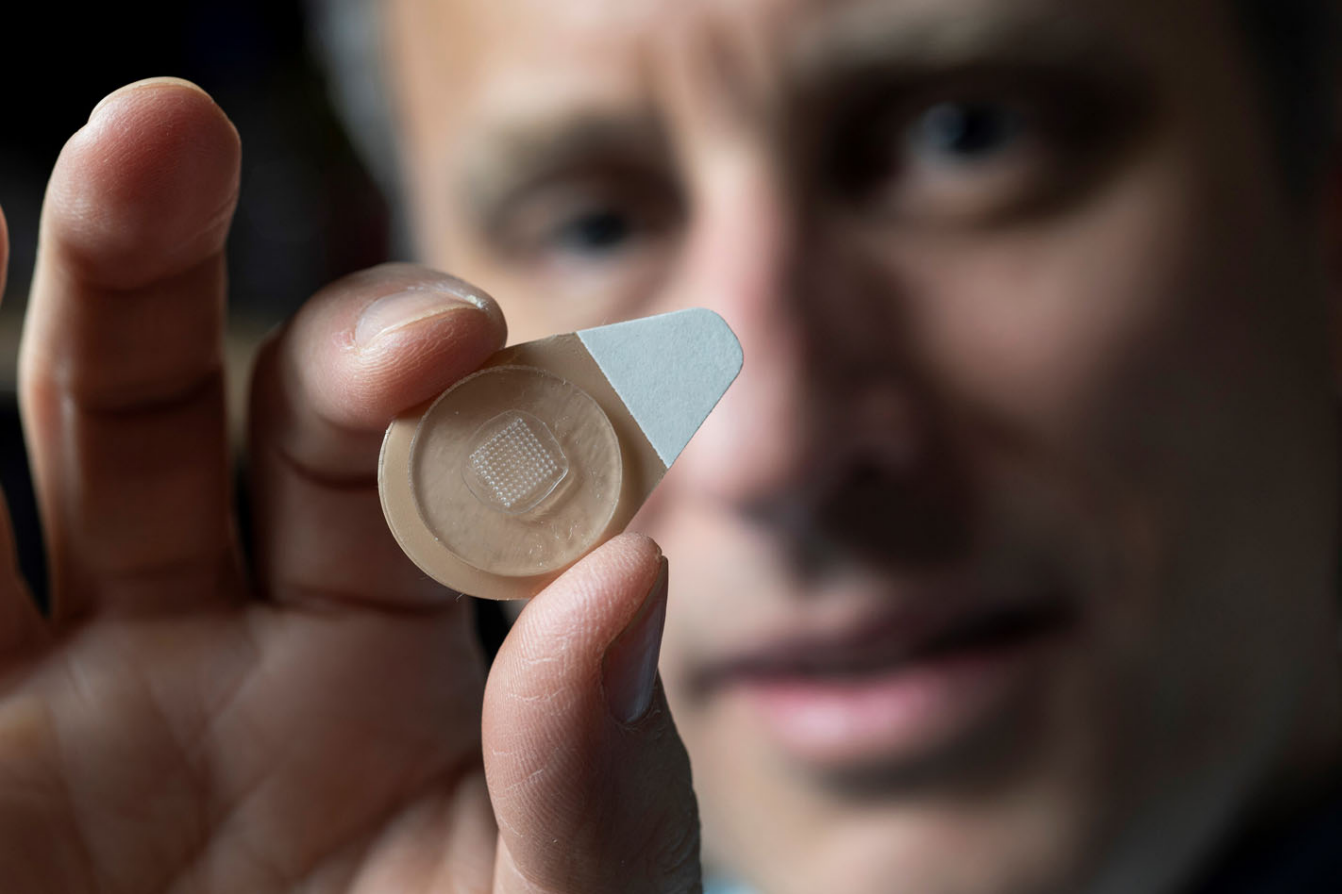
Prof. Prausnitz holding a microneedle patch

Prof. Prausnitz is the father of the “microneedle technology,” a technology that he invented for painless drug delivery through the skin. In Figure 1, Prof. Prausnitz can be seen holding a microneedle patch in his hand. Prof. Prausnitz's publication in 1998 in collaboration with Prof. Mark Allen, a Professor of Electrical & Computer Engineering at Georgia Tech at that time, was the first demonstration of microneedles for drug delivery.^1^ It was a landmark paper that spurred intense research in the field, which now includes more than 1,000 journal articles and dozens of research groups. His laboratory also conceived and demonstrated fabrication of different types of microneedles.^2^ Research works from his laboratory on the four categories of microneedles, namely the “poke and patch,”^3^ “coat and poke,”^4^ “polymeric microneedles,”^5^ and “hollow microneedles”^2^ were among some of the early seminal contributions made to the field. Prof. Prausnitz also made significant contributions to the understanding of mechanics of microneedle insertion into skin,^6^ and the effect of microneedle design on pain in human volunteers.^7^ Later, his laboratory performed in vivo evaluation of microneedles in animal models and provided evidence for microneedle effectiveness for a variety of drugs and vaccines. A couple of dozen companies have now been formed around the microneedle technology both in the US and abroad, including some by Prof. Prasunitz and his former students and postdocs. Many large corporations such as 3M, Becton, Dickinson and Company (BD) and Fujifilm have also invested in R&D to develop products based on the microneedle technology. Multiple human trials have already been conducted with microneedles, and an FDA approved intradermal flu vaccine that utilizes a hollow microneedle was recently brought to market by Sanofi. There has also been an explosive growth in the use of microneedles in the cosmetic industry where microneedle‐based products are quite popular and are being sold for applications such as for combating signs of aging.

One of the biggest impacts the microneedle technology can have is on mass vaccination by providing a painless and possibly self‐administrable method of vaccination. Microneedle patches also have the potential to mitigate the generation of hazardous sharp waste and can eliminate the need for the expensive cold‐chain currently required for storage and transportation of vaccines. Prof. Prausnitz's pivotal collaborations with Emory University and CDC has allowed his laboratory to not only develop microneedles, but to also test them in the context of vaccines for influenza, polio, measles, rubella, rabies, Ebola, filoviruses and other infectious diseases. In collaboration with Emory University, Prof. Prausnitz has recently completed the first‐in‐human clinical trial of Georgia Tech's dissolvable microneedle flu vaccine patch and has demonstrated its safety and immunogenicity in humans.^8^ A microneedle‐based vaccination device that does not require cold storage can have a tremendous impact on public health, especially in the developing nations. Recognizing this, some of the world's leading organizations such as the World Health Organization (WHO), Program for Appropriate Technology in Health (PATH), and Bill and Melinda Gates Foundation, all of whom are championing equality and healthcare for the developing nations have provided research grants to Prof. Prausnitz to develop the microneedle technology for future clinical use. Prof. Prausnitz has co‐founded Micron Biomedical to commercialize this microneedle patch technology.

Looking beyond the skin as a target organ for microneedles, Prof. Prausnitz has expanded the horizon of the microneedle technology by using them in other novel ways. For example, through collaboration with Emory University he has used microneedles to deliver medications into the eye for the treatment of macular edema (i.e., inflammation in the back of the eye), as well as macular degeneration, glaucoma and other ocular diseases. This research was commercialized by creation of a startup company that Prof. Prausnitz co‐founded. Prof. Prausnitz has recently turned his attention to the design and use of microneedles for extraction of fluids and analytes from the skin for diagnostic purposes.^9^ This could one day lead to an integrated microneedle‐based monitoring and drug delivery system.

Prof. Prausnitz has also made important contributions in the field of intracellular delivery. He has used ultrasound, convective forces, microneedles, and electroporation to deliver large molecules into the cells. Using lasers, his laboratory excited carbon nanoparticles and demonstrated delivery of proteins and DNA into cells.^10^ Research into mechanistic insight of this phenomenon revealed an interplay of high temperature bubble creation and energy transfer to cell membranes, which transiently increased their permeability.

In addition to research, Prof. Prausnitz is passionate about mentoring and teaching. He has taught courses to chemical engineering undergraduate students at Georgia Tech, and has developed advanced graduate level courses on pharmaceuticals. In 2003, through a Graduate Assistance in Areas of National Need (GAANN) award from the U.S. Department of Education (and two subsequent renewals), Prof. Prausnitz established the Center for Drug Design, Development and Delivery (CD4) at Georgia Tech. The objective of CD4 was to train the next generation of pharmaceutical scientists through interdisciplinary training in engineering and physical sciences. The training includes classroom instruction and exposure to the pharmaceutical industry through plant trips, capstone projects and discussions with industry leaders.

Prof. Prausnitz is a gifted public speaker. While still a freshman pursuing his BS in Chemical Engineering at Stanford University, Prof. Prausnitz began teaching as an instructor in the Technical Communications Program at Stanford. There he team‐taught quarter‐long classes on public speaking to undergraduate and graduate students, and continued to do so until his graduation. He then taught public speaking at MIT during his PhD, and then later at Georgia Tech after he joined as a faculty member. As a former PhD student in his laboratory, I remember Prof. Prausnitz teaching us public speaking skills during lab meetings to enable us to improve our presentation skills. Even today, he conducts one‐on‐one practice sessions with his lab members to train them for their oral presentations before they go present at conferences. The training I received from him on public speaking and communication has immensely helped me in my own career as a faculty member. Prof. Prausnitz has always emphasized simplicity and clarity in communication and encourages the use of figures and pictures over words. When I started my career as a new faculty at Texas Tech University, I was able to integrate this idea into my grant writing style, which helped me to write better research proposals.

Prof. Prausnitz's accomplishments have been recognized through numerous honors and awards including the Outstanding Work in Transdermal Drug Delivery Award and the Outstanding Pharmaceutical Paper Award both by the Controlled Release Society (CRS), CAREER Young Investigator Award by the NSF, Top 100 Young Innovator (TR100) Award by the Technology Review Magazine, NSF/NIH Scholar‐in‐Residence at the NIH Award by the NSF, Curtis W. McGraw Research Award by the ASEE, Young Investigator Award by the CRS, Outstanding Achievement in Research Program Development by Georgia Tech, Gold Tower Award: Faculty Communicator of the Year by Georgia Tech, and Highly Cited Researcher Award in 2014 and 2016 by Thomson Reuters. For his outstanding achievements, Prof. Prausnitz was named the J. Erskine Love, Jr. Chair in Chemical & Biomolecular Engineering by Georgia Tech. Prof. Prausnitz is also a member of the College of Fellows of the Controlled Release Society and AAPS.

Prof. Prausnitz is an entrepreneur at heart and has co‐founded five startup companies to commercialize discoveries and inventions from his lab, one of which is under review at the FDA for possible licensure. His expertise is widely sought in the business community and the general community, and he frequently serves as a consultant, advisory board member and an expert witness. Prof. Prausnitz has numerous patents to his name and is a fellow of the National Academy of Inventors. For his translational science and innovative research, he has been honored with the Outstanding Achievement in Research Innovation Award by Georgia Tech and Innovation Award by Georgia BIO. He was also named the Business Person of the Year (Startups to Watch Award) by Metro Atlanta Chamber of Commerce.

**Figure 2:**
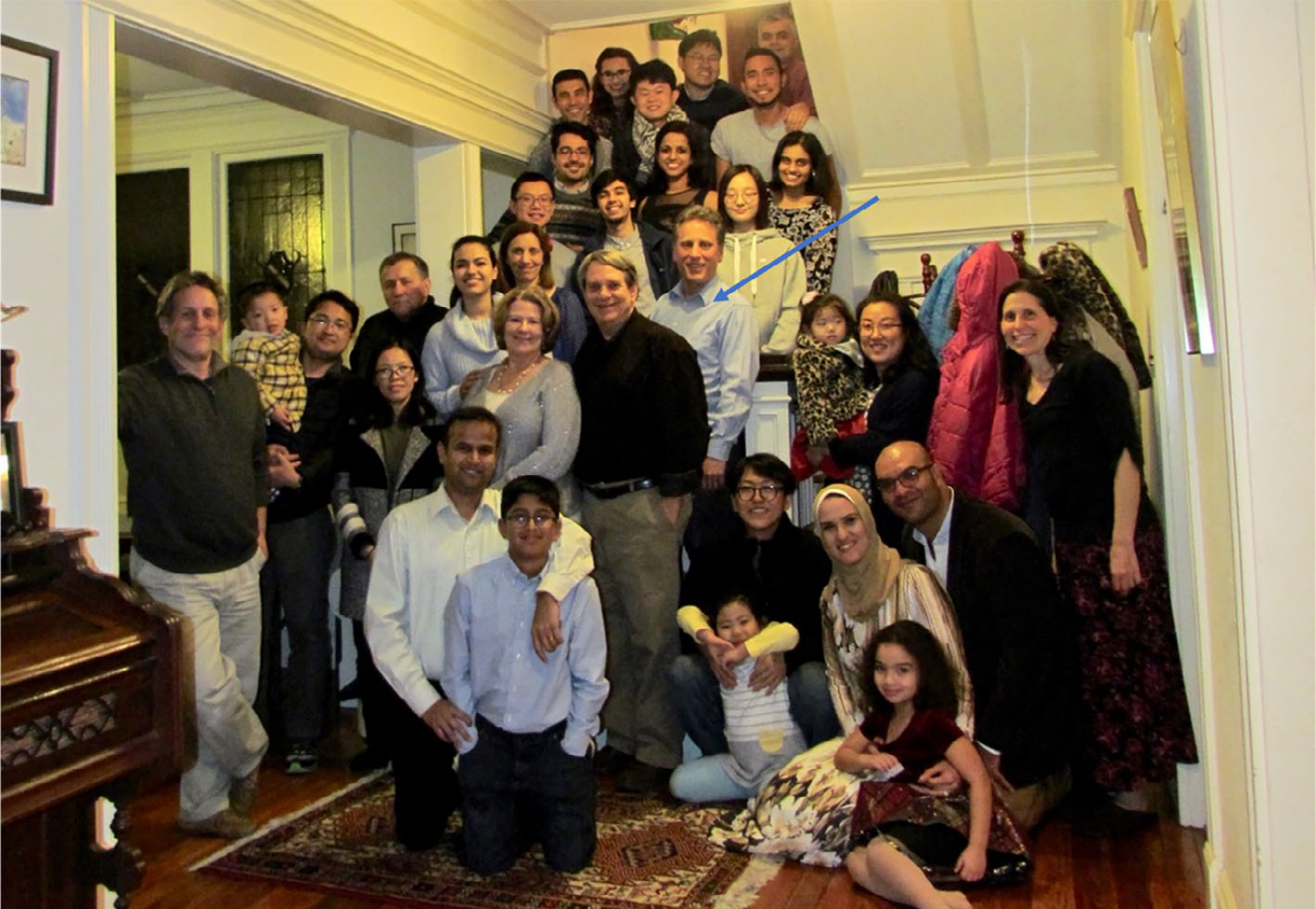
Prof. Prausnitz (indicated by arrow) with lab members and their families at a holiday party in his home

Prof. Prausnitz's work has been disseminated through more than 270 peer‐reviewed publications and he has presented over 250 invited lectures both nationally and internationally. Many of his former PhD students are working in the industry or are faculty members in universities in the US and abroad. Prof. Prausnitz's passion for innovative research, teaching, attention to detail, open door policy, and friendly nature have influenced all of his students, mentees, and trainees. Every year to celebrate the holiday season, Prof. Prausnitz throws a holiday party at his home. Figure 2 is a photo of a recent gathering in his home. My PhD years with Prof. Prausnitz were incredibly stimulating because of the outstanding and collaborative research environment he creates, and the opportunities for personal growth that naturally arise in his laboratory due to his prolific research enterprise. As a former PhD student of Prof. Prausnitz, and on behalf of all the former Prausnitz lab members I express my sincerest gratitude to Prof. Prausnitz for his guidance, training, and leading by example.
